# Circulating Tumor DNA Profiling From Breast Cancer Screening Through to Metastatic Disease

**DOI:** 10.1200/PO.20.00522

**Published:** 2021-11-24

**Authors:** Karen Page, Luke J. Martinson, Daniel Fernandez-Garcia, Allison Hills, Kelly L. T. Gleason, Molly C. Gray, Amelia J. Rushton, Georgios Nteliopoulos, Robert K. Hastings, Kate Goddard, Charlotte Ions, Vilas Parmar, Lindsay Primrose, Mark R. Openshaw, David S. Guttery, Carlo Palmieri, Simak Ali, Justin Stebbing, R. Charles Coombes, Jacqueline A. Shaw

**Affiliations:** ^1^ Leicester Cancer Research Centre, University of Leicester, Leicester, United Kingdom; ^2^ Department of Surgery and Cancer, Imperial College London, Hammersmith Hospital Campus, London, United Kingdom; ^3^ Institute of Translational Medicine, University of Liverpool, Liverpool, United Kingdom

## Abstract

**PURPOSE:**

We investigated the utility of the Oncomine Breast cfDNA Assay for detecting circulating tumor DNA (ctDNA) in women from a breast screening population, including healthy women with no abnormality detected by mammogram, and women on follow-up through to advanced breast cancer.

**MATERIALS AND METHODS:**

Blood samples were taken from 373 women (127 healthy controls recruited through breast screening, 28 ductal carcinoma in situ, 60 primary breast cancers, 47 primary breast cancer on follow-up, and 111 metastatic breast cancers [MBC]) to recover plasma and germline DNA for analysis with the Oncomine Breast cfDNA Assay on the Ion S5 platform.

**RESULTS:**

One hundred sixteen of 373 plasma samples had one or more somatic variants detected across eight of the 10 genes and were called ctDNA-positive; MBC had the highest proportion of ctDNA-positive samples (61; 55%) and healthy controls the lowest (20; 15.7%). *ESR1*, *TP53*, and *PIK3CA* mutations account for 93% of all variants detected and predict poor overall survival in MBC (hazard ratio = 3.461; 95% CI, 1.866 to 6.42; *P* = .001). Patients with MBC had higher plasma cell-free DNA levels, higher variant allele frequencies, and more polyclonal variants, notably in *ESR1* than in all other groups. Only 15 individuals had evidence of potential clonal hematopoiesis of indeterminate potential mutations.

**CONCLUSION:**

We were able detect ctDNA across the breast cancer spectrum, notably in MBC where variants in *ESR1*, *TP53*, and *PIK3CA* predicted poor overall survival. The assay could be used to monitor emergence of resistance mutations such as in *ESR1* that herald resistance to aromatase inhibitors to tailor adjuvant therapies. However, we suggest caution is needed when interpreting results from a single plasma sample as variants were also detected in a small proportion of HCs.

## INTRODUCTION

Breast cancer is the most common cancer in women worldwide, with more than 600,000 people dying annually largely because of metastatic recurrence occurring more than 5 years after diagnosis.^1^ Up to 30% of patients will relapse, and the risk of developing distant metastases potentially spans decades.^2^ Current imaging methods such as mammography and positron emission tomography–computed tomography scans have limited sensitivity and accuracy,^3^ so following a standard regime of adjuvant treatment, further screening to enable detection of micrometastases could significantly reduce the risk of recurrence and may be clinically beneficial.

**CONTEXT**

**Key Objective**
To evaluate the prevalence of circulating tumor DNA (ctDNA) in blood samples from women with early- and late-stage breast cancer, using the commercially available Oncomine Breast cfDNA Assay and explore, to our knowledge, for the first time the spectrum of mutations detected in healthy women with no evidence of any breast lesion by mammogram.
**Knowledge Generated**
Mutations in *ESR1*, *PIK3CA*, and *TP53* genes accounted for 93% of all observed variants detected across 10 breast cancer driver genes. The frequency of ctDNA-positive samples was significantly higher in patients with metastatic disease compared with healthy controls, with overall survival significantly worse in patients with detectable ctDNA.
**Relevance**
The assay accurately detects key breast cancer mutations of clinical importance such as in *ESR1* and *PIK3CA*, and could be used to stratify use of targeted therapies and monitor emergence of resistance mutations such as in *ESR1* to tailor adjuvant therapy.

Recent studies have shown the utility of circulating tumor DNA (ctDNA)-based blood tests for detecting minimal residual disease and predicting relapse.^4-6^ This ctDNA, a minor fraction of the total circulating cell-free DNA (cfDNA; < 0.1%-10%),^7^ reflects the mutational signature of the primary tumor and heterogeneity of metastatic lesions. We recently used patient-specific ctDNA analysis to detect preclinical metastases across breast cancer subtypes up to two years ahead of imaging, providing a possible window for therapeutic intervention.^8^

In patients with estrogen receptor (ER)-positive breast cancer, following prolonged exposure to adjuvant endocrine therapy, resistance mutations can emerge on metastatic progression.^9^ This is particularly true for aromatase inhibitors, given as first-line treatment in postmenopausal women with ER-positive breast cancer^10^ where around 30% of these cancers acquire activating *ESR1* gene mutations that confer resistance to treatment.^11^ With endocrine therapy considered as effective as chemotherapy,^12^ it is a preferred treatment by many women, given the lower incidence of adverse side effects. It is therefore important to develop reliable and sensitive methods to detect these acquired mutations enabling a switch in treatment at an earlier stage.

There are multiple commercially available mutation panels for ctDNA profiling; however, there is a lack of standardization and no agreement on a defined methodology.^13^ Importantly, raw data arising from ctDNA deep sequencing studies can include sequencing artifacts, germline variants, and occasional variants derived from the expansion of clonal populations of bloods cells, known as clonal hematopoiesis of indeterminate potential (CHIP).^14^ As cfDNA is derived from blood, potential CHIP mutations should be evaluated and excluded by comparison with matched germline DNA (gDNA).^15^

Here, we report the interim analysis of the first 187 samples from the Breast Screening and Monitoring Study (BSMS, IRAS 118701) and compare results with a symptomatic breast cancer cohort. The BSMS study is looking at samples from women who have been recalled after a screening mammogram, to see whether blood tests could be used to help screen for and monitor breast cancer. We tested a single blood sample from 373 women, including 127 healthy controls (HC) recruited from BSMS and 218 women with different stages of breast cancer with the Oncomine cfDNA Breast Assay. The assay enables detection of single nucleotide variants (SNVs) or insertions or deletions (INDELs) across 10 breast cancer driver genes to a variant allele frequency (VAF) of 0.1% with approximately 81% sensitivity and 99.9% specificity.^16^ We investigate the prevalence of detectable ctDNA across the disease course, define the most common mutations at each stage of BC, and explore, to our knowledge, for the first time the spectrum of mutations detected in healthy women who had no evidence of any breast lesion by mammogram. We demonstrate that the prevalence of *ESR1* and *PIK3CA* mutations increases as the disease progresses, and mutations in *ESR1*, *TP53*, and *PIK3CA* predict poor overall survival (OS) in metastatic breast cancer (MBC) and finally identify a low proportion (12.4%) of CHIP-derived mutations present.

## MATERIALS AND METHODS

### Patients and Samples

**FIG 1.**
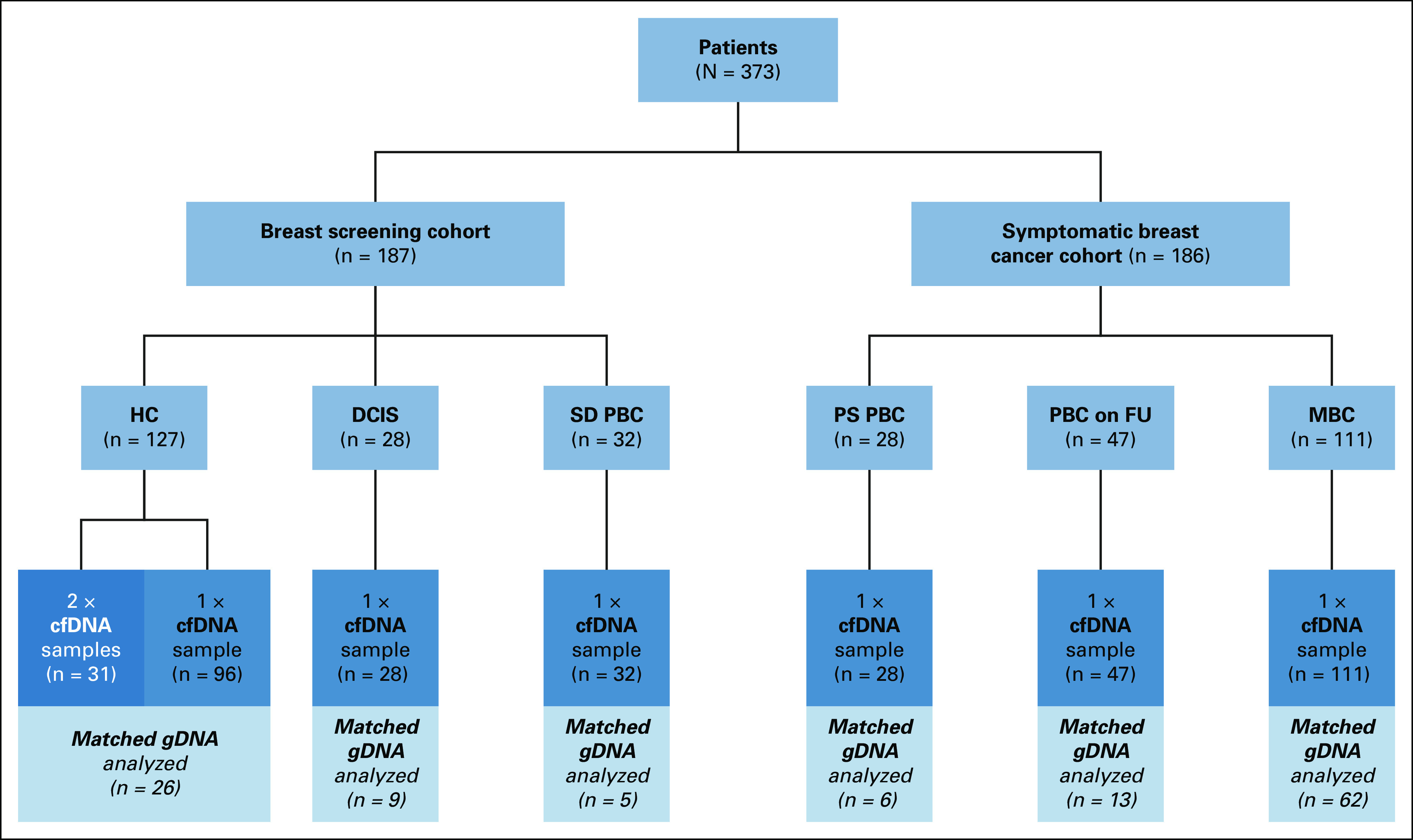
Study summary with participant groups and samples included for analysis. CONSORT diagram summarizing individuals and groups enrolled in the study, with the total number of plasma cfDNA and gDNA samples analyzed with the Oncomine Breast cfDNA Assay. Participants were from a screening cohort who were recalled because of a suspicious mammogram including HC, patients with screen detected DCIS, and patients with SD-PBC and symptomatic primary breast cancer patients who had a blood sample taken before surgery (PS-PBC), patients with primary breast cancer on follow-up, and patients with radiologically confirmed MBC. cfDNA, cell-free DNA; DCIS, ductal carcinoma in situ; gDNA, germline DNA; HC, healthy controls; MBC, metastatic breast cancer; PBC, primary breast cancer; PBC on FU, primary breast carcinoma on follow-up; PS PBC, presurgical primary breast carcinoma; SD-PBC, screen-detected primary breast cancer.

The study protocols were approved by the Riverside Research Ethics Committee (Imperial College Healthcare NHS Trust; Tissue Bank Ethics or REC reference numbers: 12/LO/2019; 13/LO/1152; R10015-16A; 07/Q0401/20), Leeds (East) MultiResearch Ethics Committee (MREC 07/H1306/164), and East Midlands Local Research Ethics Committee (REC: 13/EM/0196), conducted in accordance with Good Clinical Practice Guidelines and the Declaration of Helsinki. All 373 participants provided written informed consent before participation and were older than age 18 years. Study participants included women from a breast screening cohort and a symptomatic cohort (Fig 1, Data Supplement). Twenty milliliter blood was collected to plasma as described previously.^17^

### Extraction and Quantitation of DNA

Total cfDNA was isolated from 4 mL of plasma with the MagMAX Cell-free DNA Isolation Kit (Thermo Fisher Scientific) according to manufacturer's instructions, and gDNA was isolated from white blood cells as described previously.^18^ Quantitation and integrity checks were performed using the Qubit dsDNA BR Assay Kit (Thermo Fisher Scientific) and Agilent TapeStation HS D5000 (Agilent) according to manufacturer's instructions.

### Targeted Next-Generation Sequencing

Library reactions were set up on the Ion Chef system according to the manufacturer's protocol, using the Oncomine Breast cfDNA v1 Assay and run on either a 530 or 540 chip on the Ion S5 XL sequencing platform (all Thermo Fisher Scientific). Alignment of sequencing raw data was performed by the Torrent Suite Software version v 5.12 (Thermo Fisher Scientific). All high-confidence variant calls (those with an allele molecular coverage of ≥ 2 and Allele Mol Freq [MAF %] ≥ the limit of detection for each variant) were reviewed manually using the Integrated Genomics Viewer package (v2.3.25) by two observers.^19^

### Statistical Analysis

Mann-Whitney, Wilcoxon, and Kruskal-Wallis nonparametric tests were used to examine differences between patients regarding presence of gene mutations, and VAF of specific genes. One-way analysis of variance tests were followed by Dunn's multiple comparison test. Spearman's rank correlation coefficient was used to investigate the correlation between cfDNA quantity and the mutational VAF. These analyses were carried out using GraphPad Prism v7 software. All *P* values were two-sided and those < .05 were considered statistically significant.

Kaplan-Meier estimator and Cox regression models were used to assess OS. Survival model was constructed using the counting process notation (start, end, event)^20,21^ where the date of blood collection was taken as the start and the date of death was considered the end, with an agreed administrative censoring date of November 30, 2020. Survival curves were compared using the log-rank test. Cox proportional-hazards regression analysis was used to estimate hazard ratios for OS. Analyses were performed using R version 4.0.1, using survival (version 3.2-7) and survminer (version 0.4.8) packages.

## RESULTS

### Plasma cfDNA Levels Increase From Primary to Metastatic Disease

Here, we report the analysis of plasma cfDNA in 373 women. We performed an interim analysis in the BSMS of the first 187 women who were recalled because of an unsatisfactory mammogram, comprising 127 HC, 28 patients with ductal carcinoma in situ (DCIS), and 32 with screen-detected primary breast cancer (SD-PBC; Table 1). We compared result with 186 patients with symptomatic breast cancer including 28 unselected PBC who had a blood sample taken before surgery (PS-PBC), 47 PBC on follow-up who were receiving adjuvant therapy and were free of recurrent disease at the time of blood sampling (median follow-up time after blood sampling of 63 months [range 49-80 months]), and 111 patients with radiologically confirmed MBC (Fig 1; Data Supplement). The median age was 56 years (range of 34-91 years). One hundred ninety eight of the 218 (91%) patients with breast cancer were ER-positive, 36 (16.5%) were human epidermal growth factor receptor 2 (HER2)-positive, and 10 (4.5%) had triple-negative breast cancer (TNBC). An additional blood sample was collected from 31 of the HC to compare plasma results over time; the median time elapsed between these paired samples was 27 months (range 14-63 months; Data Supplement).

**TABLE 1.**
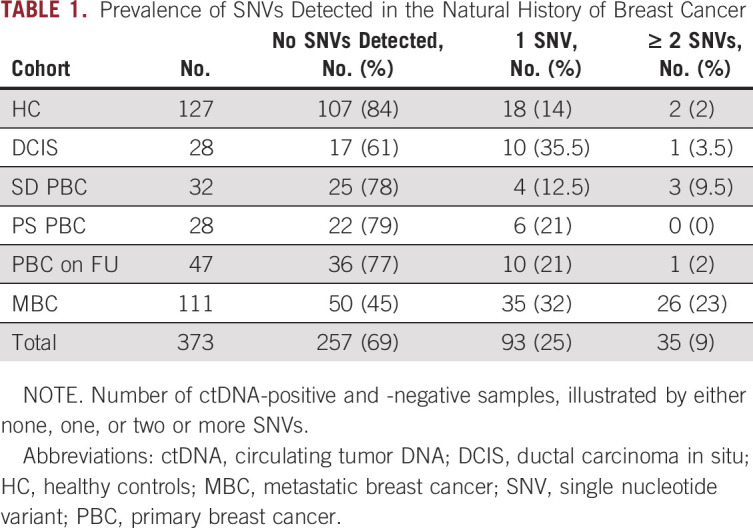
Prevalence of SNVs Detected in the Natural History of Breast Cancer

**FIG 2.**
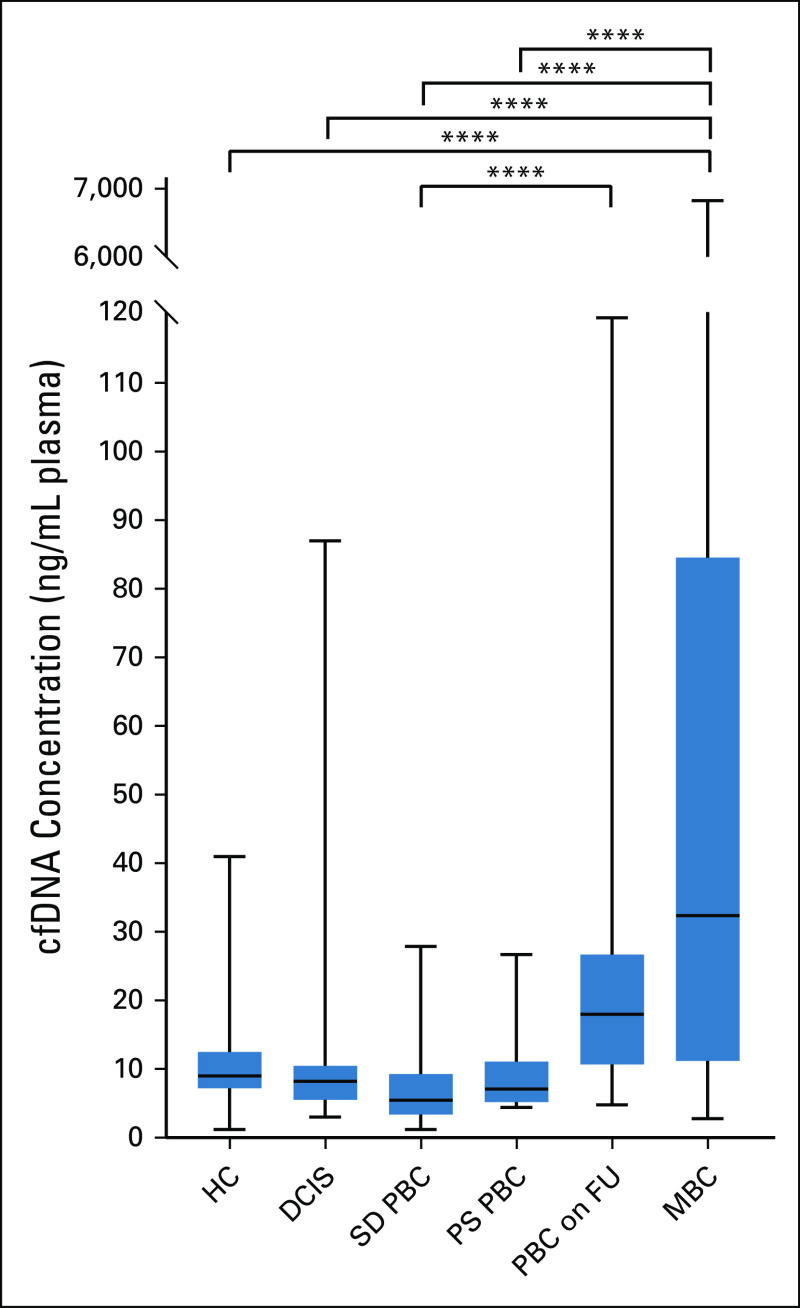
Plasma cfDNA concentration by cohort. Box and whisker plot showing plasma cfDNA concentration (ng/mL). The range, 25th and 75th percentiles, and median for each cohort (total, N = 373) are shown. HC (n = 127); DCIS (n = 28); SD PBC (n = 32); PS PBC (n = 28); PBC on FU (n = 47); MBC (n = 111). Adjusted *P* values were calculated by one-way ANOVA and Dunn's multiple comparison test. *P* values < .0001 are shown with ****; other *P* values are given in the Data Supplement. ANOVA, analysis of variance; cfDNA, cell-free DNA; DCIS, ductal carcinoma in situ; HC, healthy controls; MBC, metastatic breast cancer; PBC on FU, primary breast carcinoma on follow-up; PS PBC, presurgical primary breast carcinoma; SD PBC, screen-detected primary breast carcinoma.

Sixty-six of the 127 HC (52%) had no abnormality detected on their repeat mammogram and 61 (48%) had a core biopsy where the benign findings were predominantly of a nonproliferative nature (Data Supplement). The median plasma cfDNA concentration in HC was 9 ng/mL plasma (range 1.2-41 ng/mL) compared to 32.4 ng/mL (range 2.83-6820 ng/mL) in patients with MBC (Fig 2, Data Supplement). The HC, DCIS, SD PBC, and PS PBC groups all had cfDNA levels that were significantly lower than in MBC; the SD PBC group also had significantly lower levels than PBC FU (all adjusted *P* values < .001, Dunn's multiple comparison test, Data Supplement).

### Mutation Profiling Detects ctDNA Through the Natural History of Breast Cancer

**FIG 3.**
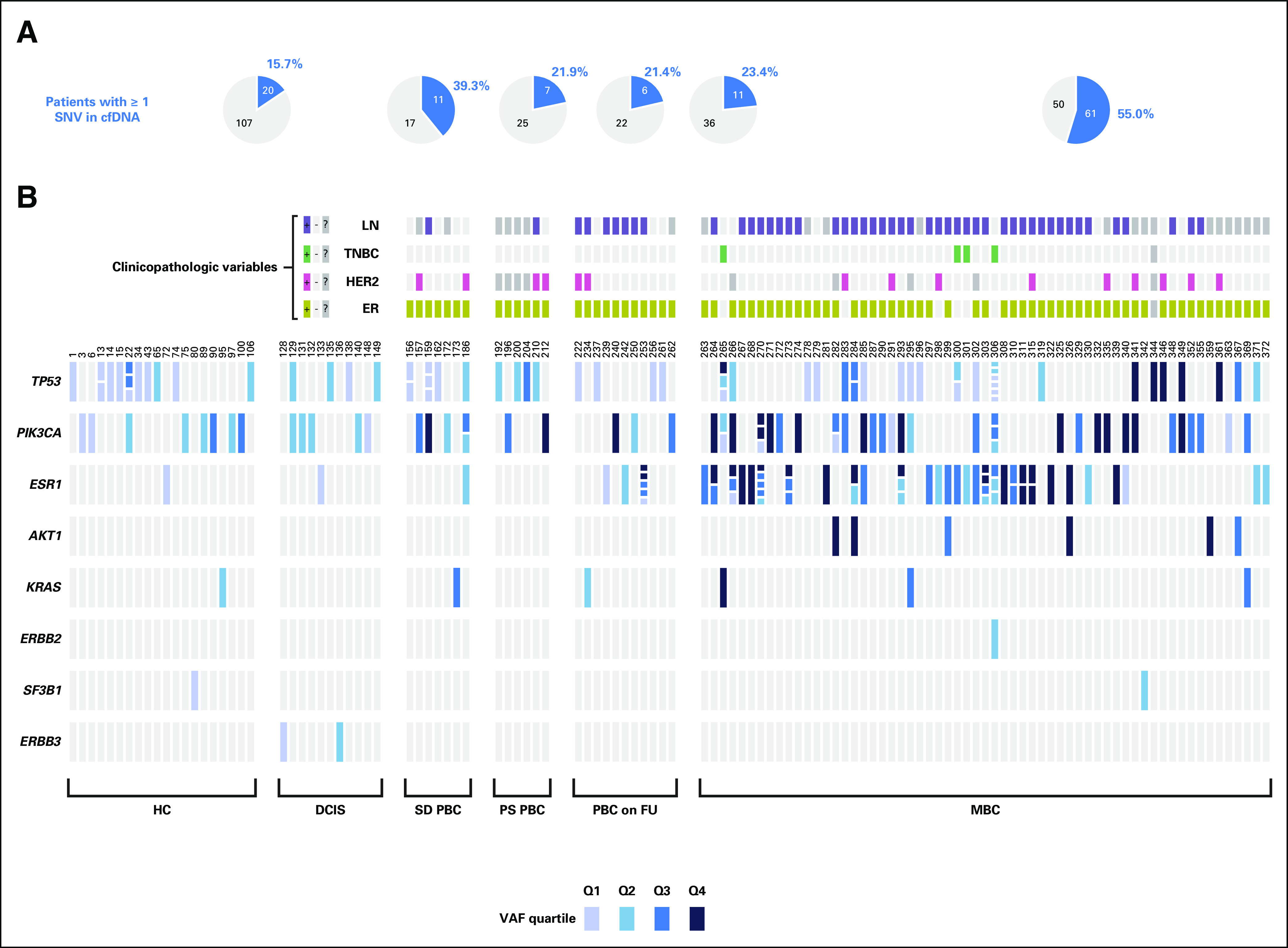
Summary of variants detected in plasma with the Oncomine Breast cfDNA Assay. (A) Pie charts showing the number of ctDNA-positive (blue) and -negative (gray) participants by cohort. (B) OncoPrint showing all ctDNA-positive samples (n = 116) based on detection of ≥ 1 SNV; blue shading indicates the VAF range of each respective SNV. SNVs were ordered according to ascending VAF and placed into uneven quartiles (Q1, n = 49, VAF range 0.06%-0.135%; Q2, n = 49, VAF range 0.142%-0.325%; Q3, n = 50, VAF range 0.33%-1.409%; and Q4, n = 49, VAF range 1.44%-52.9%) to avoid multiple variants detected at the same VAF being placed into different quartiles. In those samples where multiple SNVs were detected within the same gene, the boxes were segmented accordingly. Clinicopathologic characteristics including hormone receptor status, HER2 status, TNBC status, and LN status are indicated. ctDNA, circulating tumor DNA; DCIS, ductal carcinoma in situ; ER, estrogen receptor; HC, healthy controls; HER2, human epidermal growth factor receptor 2; LN, lymph node; MBC, metastatic breast cancer; PBC on FU, primary breast carcinoma on follow-up; PS PBC, presurgical primary breast carcinoma; Q, quartile; SD PBC, screen-detected primary breast carcinoma; SNV, single nucleotide variant; TNBC, triple-negative breast cancer; VAF, variant allele frequency.

Using a semiautomated standardized workflow and working to good clinical laboratory practice, all 404 plasma cfDNA samples and 121 gDNA samples were sequenced successfully and passed QC metrics (Data Supplement). Applying stringent thresholds, 128 of 373 (34%) first plasma cfDNA samples had one or more single nucleotide variants (SNVs) detected, and no INDELs were identified. Twelve individuals had identical variants detected in gDNA, potentially because of CHIP, and were subsequently removed from analysis. The remaining 116 plasma samples had a total of 197 SNVs detected and were classed as ctDNA-positive (Fig 3A, Data Supplement). The median VAF was 0.33% (range 0.06%-52.9%), and 140 (71%) and 86 (44%) SNVs were detected at a VAF of ≤ 1% and ≤ 0.25%, respectively (Fig 3B). Variants were detected in eight of 10 genes, notably *TP53* (31.5%), *ESR1* (30.5%), and *PIK3CA* (29.3%). No mutations were found in *EGFR* and *FBXW7*. The median VAF was *TP53* (0.12%; range 0.06%-22.03%), *ESR1* (0.62%; 0.06%-30.16%), and *PIK3CA* (0.41%; 0.06%-33.11%).

### The Number of SNVs and VAF Increase From Primary to Metastatic Disease

The frequency of ctDNA-positive samples increased from 15.7% in HC to 55% in MBC (*P* = < .001, Mann-Whitney U test; Fig 3A). Within the MBC cohort, there were 78 ER-pos and HER2-neg, 17 HER2 3+, and eight TNBC patients. Five of 28 HC with fibroadenomas and three of 22 HC with fibrocystic changes were ctDNA-positive, compared with one ctDNA-positive HC with a proliferative lesion (fibrocystic change with papillary apocrine metaplasia). In 31 of the HC, we also sought a second blood sample to compare the variant profile over time. The median time elapsed between the paired samples was 27 months (range 14-63 months). Four first and 2 second samples had one or more SNVs detected; there was no overlap, and all had low VAF (< 0.5%; Data Supplement).

Overall, 85 of the 218 (39%) patients with breast cancer were ctDNA-positive (Data Supplement) comprising 54 of 143 (37.8%) ER-pos. HER2-neg. patients, 13 of 30 (43.3%) ER pos. HER2 3+ patients, two of six ER neg. HER2 3+ patients, and four of 12 patients with TNBC. Of interest, three of the TNBC had SNVs detected in *ESR1*. A similar proportion of patients with DCIS (11 of 28 [39.3%]) were ctDNA-positive, seven of which were high grade (64%). Excluding DCIS, the number of ctDNA-positive samples was significantly lower in all groups compared with MBC (all *P* < .005; Data Supplement). Considering the 47 PBC FU patients, at the census date of November 30, 2020, two of 11 (18%) ctDNA-positive patients have relapsed, whereas only three of 36 ctDNA-negative patients (8%) have relapsed after a median follow-up of 64 months (range 50-81 months); there was no significant difference in VAF between relapsed and nonrelapsed patients. Finally, correlation analysis of cfDNA quantity and the VAF showed no significant relationship within any group (Spearman's rank correlation). When applying a higher threshold of two or more SNVs detected to define ctDNA-positive samples,^8^ there was only one ctDNA-positive sample in each of the HC, DCIS, SD-PBC, and PBC FU groups, whereas 25 (2.5%) MBCs were ctDNA-positive.

### ESR1, TP53, and PIK3CA Mutations Account for 93% of All Observed Alterations and Predict Poor Overall Survival in MBC

**FIG 4.**
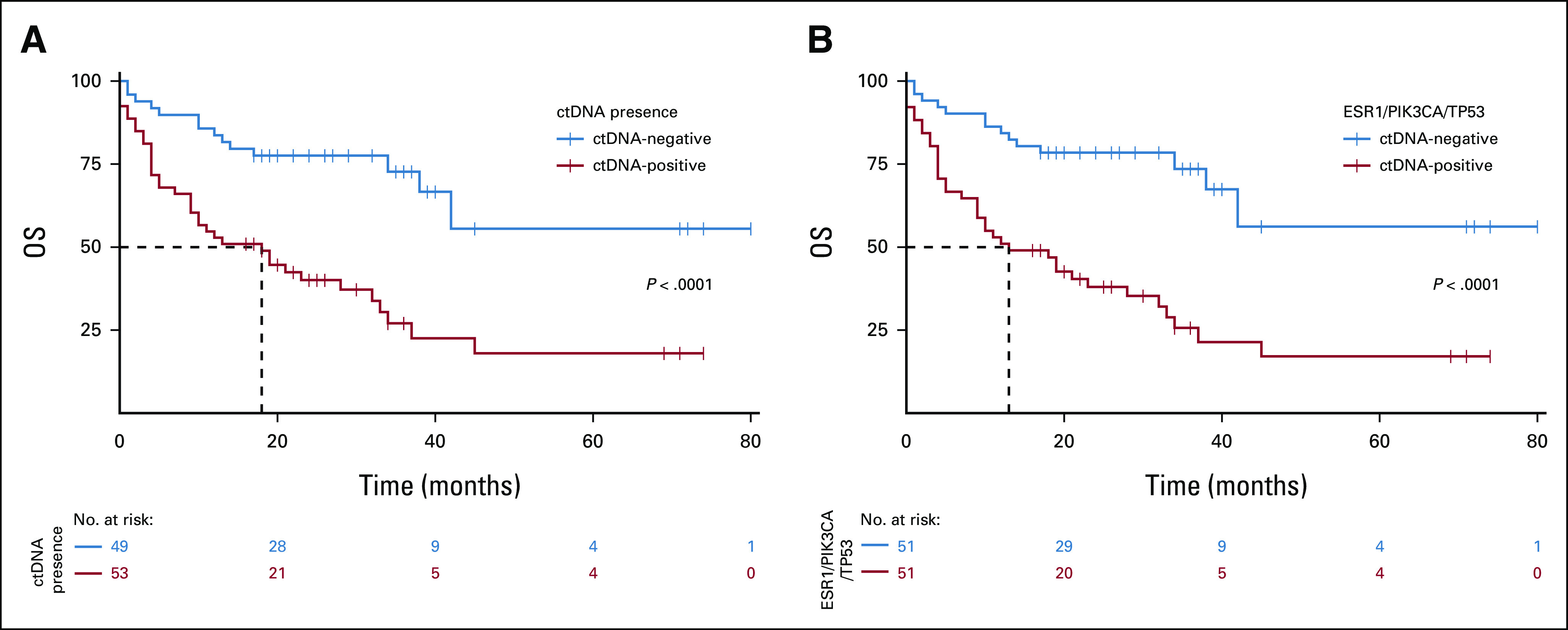
CtDNA predicts poor OS in MBC. OS analysis for MBC to explore the difference in survival outcome for ctDNA-positive patients across all genes or ctDNA-positive only in *ESR1*, *PIK3CA*, and *TP53*. Data for 100 patients with MBC; nine were removed from analysis because of incomplete information (unknown dates or state) and two were excluded with variants in other genes. (A) Median survival for ctDNA-positive patients was 18 months, whereas that for ctDNA-negative was > 80. Log-rank test *P* value < .001. Cox proportional hazards model analysis (*P* < .001) showed a hazard ratio value of 3.31 (95% CI, 1.784 to 6.142). (B) Median survival for ctDNA-positive patients (based on variants in *ESR1*, *PIK3CA*, and *TP53*) was 13 months, whereas for ctDNA-negative patients, it is > 80. Log-rank test *P* value < .001. Cox proportional hazards model analysis (*P* value < .001) showed a hazard ratio value of 3.461 (95% CI, 1.866 to 6.42). ctDNA, circulating tumor DNA; MBC, metastatic breast cancer; OS, overall survival.

SNVs within *TP53*, *ESR1*, and *PIK3CA* genes accounted for 93.4% of all variants detected. *PIK3CA* and *ESR1* SNVs were also most common in MBC and with high VAFs (Fig 3; Data Supplement). The VAF of the *TP53* variants did not differ significantly between groups; however, the frequency of *PIK3CA* variants was significantly higher in MBC than in HC (*P* = .0013; exact *P* value, Mann-Whitney U test). OS was significantly worse for patients with detectable ctDNA (hazard ratio = 3.31; 95% CI, 1.78 to 6.14; *P* < .001) and for detection of variants in *TP53*, *ESR1*, and *PIK3CA* alone (hazard ratio = 3.461; 95% CI, 1.866 to 6.42; *P* = .001; Fig 4).

### Oncomine Breast cfDNA Panel Identifies Evidence of CHIP

In 121 ctDNA-positive patients in whom we compared matched gDNA, 18 participants had an identical variant detected in their gDNA sample. Fifteen of these had variants in *TP53* and *KRAS* that were excluded as potential CHIP-derived mutations (Table 2, Data Supplement) and three of these also had other variants unique to plasma. There were three other patients with variants detected in either *PIK3CA* or *ESR1*, not previously associated with CHIP.

**TABLE 2.**
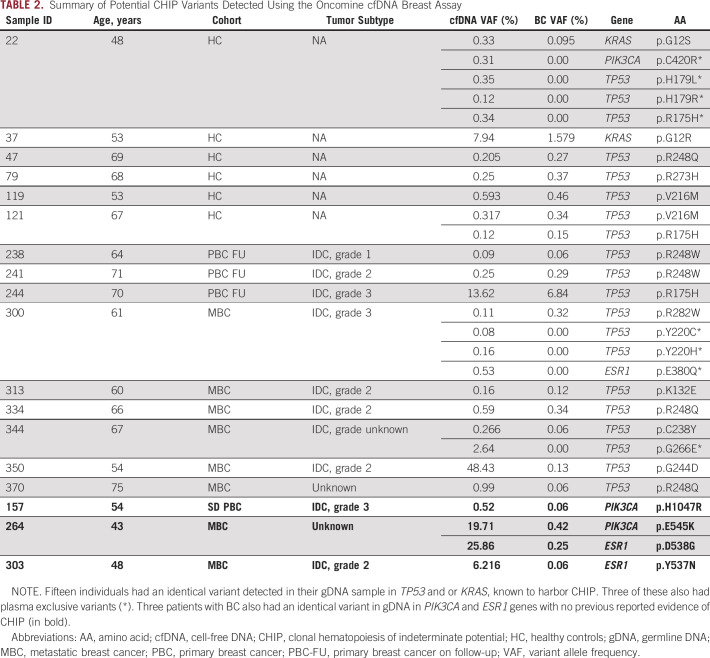
Summary of Potential CHIP Variants Detected Using the Oncomine cfDNA Breast Assay

## DISCUSSION

To our knowledge, this is the most comprehensive targeted next-generation sequencing analysis to date of ctDNA in healthy women. In 127 HC recruited through BSMS who were recalled for further investigation following suspicious mammogram, about half had no abnormality detected on their repeat mammogram and half had a core biopsy but no abnormality was detected. Overall, the majority of samples from the screening population and symptomatic PBCs were ctDNA-negative, whereas 55% of MBC were ctDNA-positive based on detection of one or more SNVs. Of interest, seven of the 11 patients with DCIS (39.3%) who were ctDNA-positive had high-grade DCIS compared with only four of 17 (24%) with lower grades; however, the number of patients with DCIS within the study is limited.

Although larger gene or mutation panels are available, as more than 93% of mutations were detected in *TP53*, *PIK3CA*, or *ESR1* genes, and also predicted poor OS in MBC, we suggest that this assay can accurately detect mutations of clinical importance, such as in *ESR1*, where emergence of mutations heralds resistance to aromatase inhibitors.^22,23^ Of interest, three patients with TNBC had *ESR1* mutations detected, suggesting the presence of a small ER-positive clone; comparison with circulating tumor cells and or metastatic tissue biopsy would be helpful in these patients to explore tumor evolution. The prevalence of *TP53* and *PIK3CA* gene mutations were similar to other studies,^24-26^ indicating that this panel is effective in identifying actionable mutations including *PIK3CA* mutations that indicate those patients who might benefit from treatment with PI3K inhibitors such as alpelisib.^27^ Our data show that variants in *ESR1* and *PIK3CA* are identified in only a small proportion of HC and become much more prevalent in MBC, indicating the presence of these mutations arise because of the evolving cancer and not because of the benign pathology of a core biopsy. In patients with MBC, as expected, the median VAF was significantly higher compared with HC, DCIS, and SD PBC, and a greater range of SNVs were detected, indicating the presence of different subclones. A small number of studies have explored the Oncomine cfDNA Assay in breast cancer; one was limited to advanced disease,^28^ whereas another was used to determine whether mutations could be identified in cfDNA from serum stored for 30 years.^29^

We also investigated the prevalence of CHIP-derived mutations in ctDNA, normal hemopoietic cells accumulating somatic mutations during the aging process in the absence of cancer.^14^ Such mutations can potentially arise in highly sensitive next-generation sequencing data because of inaccurate variant calling, sequencing artifacts, and somatic tumor–relevant SNPs. Swanton et al^30^ demonstrated that CHIP variants occur at a VAF of < 0.1% and are more common than previously documented. In 15 ctDNA-positive patients, identical variants were seen in the matched gDNA; 13 were missense, nonfunctional *TP53* mutations and two were in *KRAS*. This is in agreement with Chen and Liu^28^ who identified similar *TP53* mutations, one of the five most commonly mutated genes in CHIP. We agree with others that matched gDNA should be analyzed in parallel to exclude CHIP-derived mutations, to facilitate appropriate and accurate variant calling.^14^

We hoped that in patients who have abnormal or indeterminate mammography results, ctDNA analysis at the time of breast screening could be used to help clinicians decide whether surgery is needed. Our results suggest that this test might be useful for stratifying biopsy-confirmed screen-detected cancers to neoadjuvant therapies. However, caution is needed in interpreting ctDNA results from a single blood test at this time as we found a small proportion of HC who had detectable ctDNA, but on retesting, no individual was found to have a persistent variant profile.

Since the inception of this study, several groups have reported different assays potentially capable of helping in the differential diagnosis of breast diseases, and thus are candidate blood tests for use as a part of a screening strategy. These include DNA methylation analysis,^29^ ctDNA fragmentation assays,^22^ and a multianalyte platform.^23^ Our results show that screening with the assay could be used in combination with other such strategies, and future studies could focus on developing technologies to improve sensitivity, determining the added value of measuring other analytes,^23^ and the application of machine learning^22^ in improving diagnostic accuracy.

In conclusion, the assay successfully detected key breast cancer mutations through ctDNA and predicted poor OS in MBC. Further investigation is needed regarding the prevalence of mutations in healthy women in a true cancer screening setting to attempt to define a ctDNA baseline in the healthy population.

## References

[1] Ferlay J Colombet M Soerjomataram I et al Estimating the global cancer incidence and mortality in 2018: GLOBOCAN sources and methods Int J Cancer 144:1941–1953 2019 30350310 10.1002/ijc.31937

[2] Pan H Gray R Braybrooke J et al 20-Year risks of breast-cancer recurrence after stopping endocrine therapy at 5 years N Engl J Med 377:1836–1846 2017 29117498 10.1056/NEJMoa1701830 PMC5734609

[3] Fenton JJ Taplin SH Carney PA et al Influence of computer-aided detection on performance of screening mammography N Engl J Med 356:1399–1409 2007 17409321 10.1056/NEJMoa066099 PMC3182841

[4] Garcia-Murillas I Schiavon G Weigelt B et al Mutation tracking in circulating tumor DNA predicts relapse in early breast cancer Sci Transl Med 7:302ra133 2015 10.1126/scitranslmed.aab0021 26311728

[5] Olsson E Winter C George A et al Serial monitoring of circulating tumor DNA in patients with primary breast cancer for detection of occult metastatic disease EMBO Mol Med 7:1034–1047 2015 25987569 10.15252/emmm.201404913 PMC4551342

[6] Shaw JA Page K Blighe K et al Genomic analysis of circulating cell-free DNA infers breast cancer dormancy Genome Res 22:220–231 2012 21990379 10.1101/gr.123497.111 PMC3266030

[7] Diehl F Schmidt K Choti MA et al Circulating mutant DNA to assess tumor dynamics Nat Med 14:985–990 2008 18670422 10.1038/nm.1789 PMC2820391

[8] Coombes RC Page K Salari R et al Personalized detection of circulating tumor DNA antedates breast cancer metastatic recurrence Clin Cancer Res 25:4255–4263 2019 30992300 10.1158/1078-0432.CCR-18-3663

[9] Johnston SR Kilburn LS Ellis P et al Fulvestrant plus anastrozole or placebo versus exemestane alone after progression on non-steroidal aromatase inhibitors in postmenopausal patients with hormone-receptor-positive locally advanced or metastatic breast cancer (SoFEA): A composite, multicentre, phase 3 randomised trial Lancet Oncol 14:989–998 2013 23902874 10.1016/S1470-2045(13)70322-X

[10] Allouchery V Beaussire L Perdrix A et al Circulating ESR1 mutations at the end of aromatase inhibitor adjuvant treatment and after relapse in breast cancer patients Breast Cancer Res 20:40 2018 29769099 10.1186/s13058-018-0968-0 PMC5956618

[11] Toy W Shen Y Won H et al ESR1 ligand-binding domain mutations in hormone-resistant breast cancer Nat Genet 45:1439–1445 2013 24185512 10.1038/ng.2822 PMC3903423

[12] Palmieri C Cleator S Kilburn LS et al NEOCENT: A randomised feasibility and translational study comparing neoadjuvant endocrine therapy with chemotherapy in ER-rich postmenopausal primary breast cancer Breast Cancer Res Treat 148:581–590 2014 25395314 10.1007/s10549-014-3183-4

[13] Page K Shaw JA Guttery DS The liquid biopsy: Towards standardisation in preparation for prime time Lancet Oncol 20:758–760 2019 31162088 10.1016/S1470-2045(19)30310-9

[14] Genovese G Kahler AK Handsaker RE et al Clonal hematopoiesis and blood-cancer risk inferred from blood DNA sequence N Engl J Med 371:2477–2487 2014 25426838 10.1056/NEJMoa1409405 PMC4290021

[15] Razavi P Li BT Brown DN et al High-intensity sequencing reveals the sources of plasma circulating cell-free DNA variants Nat Med 25:1928–1937 2019 31768066 10.1038/s41591-019-0652-7 PMC7061455

[16] Dhingra D Chien R Gu J et al An NGS workflow to detect down to 0.1% allelic frequency in cfDNA for breast and colon cancers Cancer Res 77 2017 abst 5396

[17] Page K Guttery DS Zahra N et al Influence of plasma processing on recovery and analysis of circulating nucleic acids PLoS One 8:e77963 2013 24205045 10.1371/journal.pone.0077963 PMC3799744

[18] Guttery DS Page K Hills A et al Noninvasive detection of activating estrogen receptor 1 (ESR1) mutations in estrogen receptor-positive metastatic breast cancer Clin Chem 61:974–982 2015 25979954 10.1373/clinchem.2015.238717

[19] Robinson JT Thorvaldsdottir H Winckler W et al Integrative genomics viewer Nat Biotechnol 29:24–26 2011 21221095 10.1038/nbt.1754 PMC3346182

[20] Andersen PK Gill RD Cox regression-model for counting-processes—A large sample study Ann Stat 10:1100–1120 1982

[21] Fernandez-Garcia D Hills A Page K et al Plasma cell-free DNA (cfDNA) as a predictive and prognostic marker in patients with metastatic breast cancer Breast Cancer Res 21:149 2019 31856868 10.1186/s13058-019-1235-8 PMC6924016

[22] Cristiano S Leal A Phallen J et al Genome-wide cell-free DNA fragmentation in patients with cancer Nature 570:385–389 2019 31142840 10.1038/s41586-019-1272-6 PMC6774252

[23] Cohen JD Li L Wang Y et al Detection and localization of surgically resectable cancers with a multi-analyte blood test Science 359:926–930 2018 29348365 10.1126/science.aar3247 PMC6080308

[24] Cancer Genome Atlas Network Comprehensive molecular portraits of human breast tumours Nature 490:61–70 2012 23000897 10.1038/nature11412 PMC3465532

[25] Curtis C Shah SP Chin SF et al The genomic and transcriptomic architecture of 2,000 breast tumours reveals novel subgroups Nature 486:346–352 2012 22522925 10.1038/nature10983 PMC3440846

[26] Martinez-Saez O Chic N Pascual T et al Frequency and spectrum of *PIK3CA* somatic mutations in breast cancer Breast Cancer Res 22:45 2020 32404150 10.1186/s13058-020-01284-9 PMC7222307

[27] Andre F Ciruelos E Rubovszky G et al Alpelisib for *PIK3CA*-mutated, hormone receptor-positive advanced breast cancer N Engl J Med 380:1929–1940 2019 31091374 10.1056/NEJMoa1813904

[28] Chen S Liu Y p53 involvement in clonal hematopoiesis of indeterminate potential Curr Opin Hematol 26:235–240 2019 31045645 10.1097/MOH.0000000000000509 PMC12050009

[29] Delmonico L Silva Magalhães Costa MA Gomes RJ et al Methylation profiling in promoter sequences of *ATM* and *CDKN2A* (*p14*^*ARF*^/*p16*^*INK4a*^) genes in blood and cfDNA from women with impalpable breast lesions Oncol Lett 19:3003–3010 2020 32218857 10.3892/ol.2020.11382 PMC7068365

[30] Swanton C Venn O Aravanis A et al Prevalence of clonal hematopoiesis of indeterminate potential (CHIP) measured by an ultra-sensitive sequencing assay: Exploratory analysis of the Circulating Cancer Genome Atlas (CCGA) study J Clin Oncol 36 2018 suppl; abstr 12003

